# Molecular epidemiology survey and characterization of human influenza A viruses circulating among Palestinians in East Jerusalem and the West Bank in 2015

**DOI:** 10.1371/journal.pone.0213290

**Published:** 2019-03-08

**Authors:** Maysoon Bakri, Monjed Samuh, Maysa Azzeh

**Affiliations:** 1 Virology Research Laboratory, Medical Research Center, Al-Quds University, Abu Dies-East Jerusalem, West Bank, Palestine; 2 Department of Applied Mathematics and Physics, Palestine Polytechnic University, Hebron, West Bank, Palestine; Sun Yat-Sen University, CHINA

## Abstract

Frequent typing and molecular characterization of influenza A (IAV) strains are crucial for the identification of circulating subtypes and for the selection of the subtypes’ lineages to be included in the annually prepared vaccine cocktail. We investigated IAV sampled from an underrepresented population from Palestine. 200 nasopharyngeal aspirates (NPA) were collected between February and May of 2015 from Palestinians in East Jerusalem and the West Bank suffering from mild to severe symptoms of upper respiratory infections. NPA were screened for the presence of IAV using RT-PCR. Epidemiological data, hemagglutinin (HA) and neuraminidase (NA) gene sequences were analyzed in IAV positive samples. 50 samples tested positive for IAV; 48% of which were identified as A(H1N1)pdm09 and 52% as A(H3N2), respectively. Infection with A(H1N1)pdm09 occurred mainly in April, while A(H3N2) infections were mainly detected in March. Most IAV infections in 6-year-olds and below were attributed to subtype A(H3N2), while A(H1N1)pdm09 was responsible for most infections in adults above 18-year-olds. Analyses of HA and NA amino acid sequences revealed numerous substitutions. Thereafter, and based on the HA analysis, the Palestinian A(H1N1)pdm09 isolates fell into clade 6B, while the A(H3N2) isolates fell into clades 3C.2 and 3C.3, respectively. This study is significant in providing the first insight into the epidemiology and genetic properties of IAV circulating in Palestine. In contrast to international reports for the same season, A(H3N2) was not the dominant subtype as in northern hemisphere, nor was A(H1N1)pdm09 as in WHO reports for the Middle East, however genetic properties of Palestinian A(H3N2) and A(H1N1)pdm09 were in line with global isolates.

## Introduction

Influenza A virus (IAV) infections cause significant mortality, morbidity, and socio-economic burden throughout the world. One of the diseases described in the ancient history of medicine by Hippocrates in 412 BC is believed to be influenza infection [1]. The first recognized influenza pandemic was in 1510 [2, 3]. Thereafter, another nine epidemics were identified prior to the well-known “Spanish” influenza pandemic of 1918/1919 [2, 3], the causative influenza virus strain of which was only identified and characterized definitely in 1997 [4, 5].

In contrast to many other vaccines, influenza vaccines have to be evaluated and updated annually due to antigenic shift and drift. For example, variability of the circulating A(H3N2) subtype has required four changes in the vaccine component between 2014 and 2018 [6]. Vaccines against influenza are trivalent and contain one A(H1N1), one A(H3N2), and one influenza B strain or quadrivalent and contain one A(H1N1), one A(H3N2), and two influenza B strains. These are predicted as most likely to circulate in the following season. While WHO usually recommended trivalent influenza vaccine, recommendation for 2018/2019 influenza season was to use a quadrivalent vaccine [6]. This decision was made as a consequence of the 2017/2018-influenza season with the majority of circulating influenza B belonging to the B/Yamagata/16/88 lineage; in Europe specifically the majority of influenza infections in 2018 of season 2017/2018 were caused by Influenza B viruses [7].

This report describes influenza A viruses circulating in Palestine in 2015, a country with very limited surveys in the epidemiology and genetic properties of infectious agents. Influenza season 2014/2015 was a season featured with low vaccine efficacy against the A(H3N2) vaccine component (A/Texas/50/2012), which consequently led to its replacement by A/Switzerland/9715293/2013 [8, 9]. Molecular characterization of circulating influenza viruses is indispensible for the annual vaccine recommendation. To the best of our knowledge, this is the first report tackling molecular epidemiology and characterization of human influenza A viruses circulating in Palestine.

## Materials and methods

### Ethics statement

The Al-Quds University ethical committee approved this study (8/REC/16 in 2014 and 4/REC/19 in 2015). Al-Makassed Islamic Charitable Hospital (MICH), the referral hospital for Palestine, two kindergartens, and a private clinic approved sampling in their entity. Every single participant signed a consent form before sampling, with parents or legal guardians signing for children. Consent form contained the following participants’ data: sex, age, residency, symptoms, hospitalized days, vaccination status, and administration of antibiotics.

### Study population and collection of nasopharyngeal aspirate

The study population included Palestinian children and adults from East Jerusalem and the West Bank that suffered mild to severe upper respiratory infection symptoms. Participants were either hospitalized, outpatients, kindergarten children, family members and acquaintance (referred to as private in the manuscript), or visiting a private clinic. Nasopharyngeal aspirate (NPA) were collected using FLOQSwabs^TM^ (#502CS01, Copan Diagnostics Inc., USA).

### Influenza A RNA extraction and RT-PCR

Influenza A RNA was extracted from the nasopharyngeal aspirate using the QIAamp Viral RNA Mini kit (Qiagen, Hilden, Germany). Amplification of the hemagglutinin (HA) and neuraminidase (NA) genes occurred in a one step RT-PCR reaction using One-Step RT-PCR Kit (Qiagen, Hilden, Germany). Complete HA and NA ORFs were basically amplified using primer sets recommended by WHO [10]. Hereby, two primer pairs were used to amplify each segment. The primer pair H3N2R1104 and N2F257 for A(H3N2) amplification were modified during this research based on initial sequencing results to enhance the yield of the RT-PCR product as following H3N2R1104 (ATCCACACGTCATTTCCATCATCA) and N2F257 (AAACCAGCAGAATACAGAAATTGGTC). Screening for A(H1N1)pdm09 and A(H3N2) was performed using the N1F401/NARUc and H3A1F3/HARUc primer pairs respectively [10].

### Sequence analysis and typing

Sequences were aligned with NCBI- and GISAID-archived [11] HA and NA sequences (S1 Table), using MegAlign program (Lasergene, version 14, DNASTARInc., WI, USA). The Lasergene MegAlign software marks nucleotide differences between the aligned sequences. Subsequently, chromatograms of our sequences were inspected visually for these nucleotides to check if the chromatogram peak and deduced bases match. This procedure is essential to verify whether a detected nucleotide substitution was due to real mutation or to a technical problem in the automatic chromatogram base calling. The annotated Palestinian sequences were submitted to GenBank and assigned the accession numbers KY075819-KY075852.

### Substitution analysis

Substitution events in the Palestinian HA and NA genes were identified by comparing sequences to the A(H1N1)pdm09 and A(H3N2) vaccine strains of the isolation season 2014/2015, A/California/07/2009(H1N1) and A/Texas/50/2012(H3N2). Occurrence of these substitutions in archived sequences (S1 Table) and recent vaccine strains (A/Michigan/45/2015(H1N1), A/Switzerland/9715293/2013(H3N2), and A/Hong Kong/4801/2014(H3N2)) was also recorded. Substitution analysis was performed using MegAlign program. Additionally, amino acid substitution/mutation report provided by FluServer [12] was used to assess circulation of the substitution. To delineate substitutions, PubMed and FluServer [12] databases were queried. Information regarding location of substitution within antigenic sites of A(H3N2) isolates was identified based on epitope mapping reports [13–15]. Substitution residues in the antigenic sites of A(H1N1)pdm09 isolates were identified based on recent studies, which used A/California/07/2009 for mapping epitopes [16–18].

We noticed that the HA substitutions’ numbering in the literature was not consistent, as some authors used the amino acid (aa) position on the HA protein while others used aa positions on the HA1 and HA2 subunits. Therefore, we decided to indicate both position numberings in our tables to avoid confusion; however, we used the HA1 and HA2 substitution numbering throughout the manuscript and underlined the HA2 substitutions to distinguish them from HA1 ones.

### Phylogenetic trees

In MegAlign program aligned Palestinian and reference sequences (S1 Table) were used to create phylogenetic trees. Phylogenetic trees were generated in Mega 7 program (https://www.megasoftware.net) using Maximum likelihood test, HKY+G model, and with 1,000 bootstrap replicates; only bootstrap values over 50 were shown.

## Results

### Study sample data and demographics

200 NPA samples were collected between February and May of 2015 distributed as 9.5% in February, 48% in March, 33.5% in April, and 9% in May. Most of the NPA samples were collected from kindergarten (48%), with an almost equal number of samples collected from hospitalized patients/outpatients (17.5%), private clinic patients (17%), and private (17.5%). The study sample was divided into four age groups: infants (≤1 year), children before school age (1–6 years), school age (7–18 years), and adults (>18 years). Their distribution percentages were 11.5%, 63%, 5% and 20.5% respectively. As for sex of the study sample, 58.5% were males and 41.5% were females. The majority of study sample was from East Jerusalem (75.5%), while 24.5% was from the West Bank.

Runny nose was the most common symptom among the participants (76.5%), followed by cough (42.2%) and fever (21.4%). Single cases presented with sore throat, diarrhea, vomit, ear infection, bronchitis, chest pain, shortness of breath, and sputum discharge.

### Influenza A positive samples

50 samples (25%) of the collected 200 NPA tested positive for IAV, from which 24 represented A(H1N1)pdm09, and 26 represented A(H3N2) subtype, respectively. IAV infection distributed almost equally between males (54%) and females (46%), while subtype H3N2 was responsible for most IAV infections among males (65.4%), H1N1 caused more infections in females (58.3%); this was not statistically significant.

Antibiotics were administrated in 23 cases, 6 of which tested positive for IAV (3 H1N1 and 3 H3N2). In six cases, patients received antibiotics and Tamiflu or Tamiflu alone, none of them tested positive for IAV. As only 15 of the study sample consented to have received the flu shot, four of which tested positive for IAV (2 H1N1 and 2 H3N2), we were unable to make a statement about significance in that regard.

**Fig 1:**
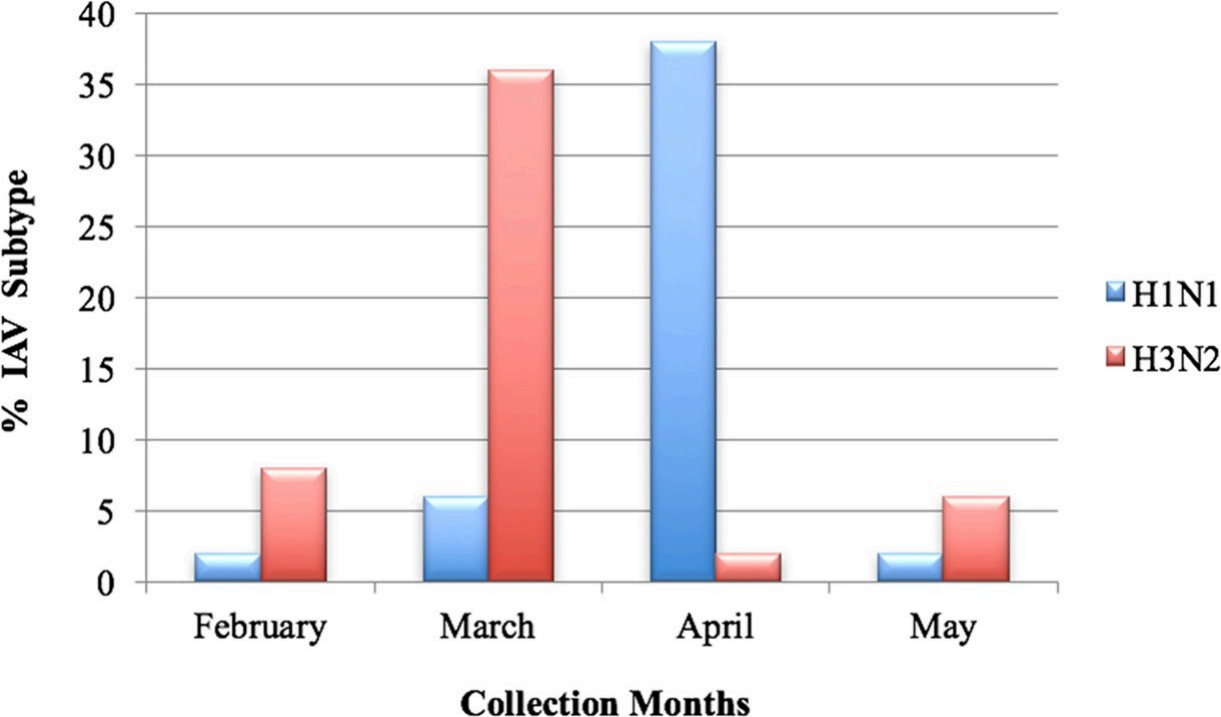
Distribution of IAV subtypes in the months of collection. The percentages reflect the numbers of each subtype in each month in relevance to the 50 total positive IAV cases.

While the monthly distribution of IAV positive samples relative to collected samples each month did not significantly vary between the months (26.3% on February, 21.8% on March, 29.9% on April and 22.2% on May), the monthly distribution of the subtypes changed. In fact, the majority of the A(H1N1)pdm09 cases were detected in April, while the majority of the A(H3N2) cases were detected in March (Fig 1). Fisher’s Exact Test for count data was performed to test whether being infected by A(H1N1)pdm09 and A(H3N2) is associated with the month of sampling. The test was significant for both subtypes (p-value = 0.00019 for A(H1N1)pdm09 and 0.0011 for A(H3N2)).

**Fig 2:**
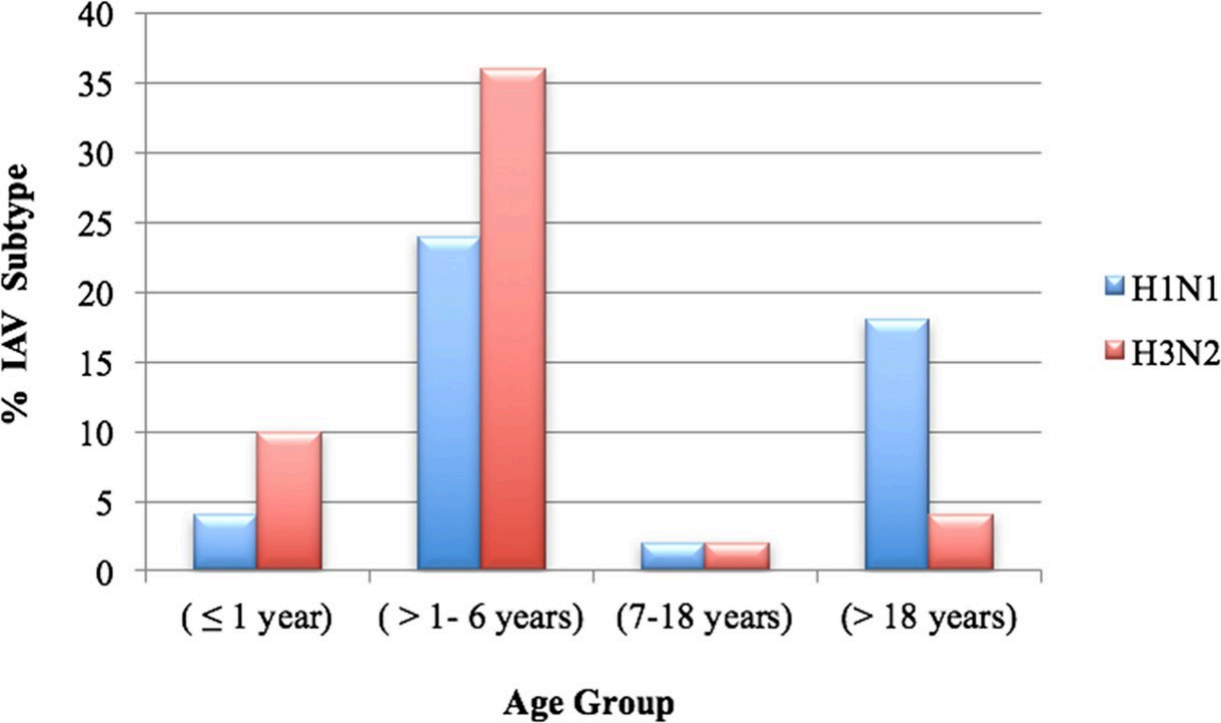
Distribution of A(H1N1)pdm09 and A(H3N2) subtypes in the different age groups. The distribution of IAV subtypes throughout the different age groups was calculated relative to the 50 total positive IAV samples.

The percentages of cases infected with IAV throughout the four age groups relative to the number of total samples presented in each group were 30.4%, 23.8%, 20%, and 26.8% for age group ≤1 year, 1–6 years, 7–18 years, and >18 years, respectively. However, the distribution of IAV subtypes throughout the different age groups varied. Most of the IAV infections in the eldest age group (>18 years) were due to A(H1N1)pdm09 (Fig 2). Infections with A(H1N1)pdm09 and A(H3N2) were equal in the age group 7–18 years, while most of the IAV infections in the youngest age groups; 1–6 years and ≤1 year, were caused by A(H3N2) subtype (Fig 2). Although this finding was not statistically significant based on Fisher’s exact test (p-value = 0.078 for H1N1 and 0.094 for H3N2), the test revealed that the odds of being infected with A(H1N1)pdm09 among adults were higher than the odds of being infected with A(H1N1)pdm09 in those ≤6 years, while the odds of being infected with A(H3N2) in subjects ≤6 years were higher than in adults.

### Sequencing results of HA and NA genes

HA and NA segments of all 50 positive IAV samples were subjected to gene amplification and sequencing. Complete or partial HA and/or NA sequencing were obtained in 10 H1N1 (three samples with full length HA and NA genes’ sequences, seven with ~70% partial NA sequences only) and 12 H3N2 (six samples with full length HA and NA sequences, one with full length HA and partial NA, three with full length HA only, and two with partial HA gene sequence only).

### Substitutions in the HA and NA of Palestinian A(H1N1)pdm09 isolates

Twenty non-synonymous substitutions were detected in the three HA genes of the Palestinian A(H1N1)pdm09 isolates; three occurred in the signal peptide, ten in the HA1 domain, and seven in the HA2 domain (Table 1). With the exception of A3D substitution in the signal peptide, all other substitutions occurred in NCBI- and/or GISAID-archived [11] sequences. Seven out of the ten substitutions in the HA1 domain of the Palestinian A(H1N1)pdm09 isolates; P83S, S84N, K163Q, S185T, S203T, K283E, and I295V were located within antigenic epitopes. 27 synonymous substitutions were also detected in HA of the Palestinian A(H1N1)pdm09 isolates (S2 Table). The NA gene of the A(H1N1)pdm09 isolates exhibited 20 non-synonymous (Table 2) and 21 synonymous (S3 Table) substitutions.

**Table 1:** Non-synonymous substitutions in the HA gene of Palestinian A(H1N1)pdm09 isolates (n = 3).

nt HA	aa HA	aa HA1/ HA2	Occurrence in Palestinian Sequences	Circulation of Substitution
C8A	A3D	SP	1*	No
A9C				
T28C	Y10H	SP	1	2015
G37A	A13T	SP	2*	Since 2009^**+**^
C298T	P100S	P83S	3*	Since 2009^**+**^
G302A	S101N	S84N	1	Since 2009^**+**^
G340A	D114N	D97N	3*	Since 2009
A538C	K180Q	K163Q	3*	Since 2010^**+**^
G605C	S202T	S185T	3*	Since 2009^**+**^
T658A	S220T	S203T	3*	Since 2009
G817A	A273T	A256T	3*	Since 2009^**+**^
A898G	K300E	K283E	3*	Since 2009^**+**^
A934G	I312V	I295V	1	Since 2015
A1012G	I338V	I321V	3*	Since 2009
G1171A	E391K	E47K	3*	Since 2009^**+**^
G1403A	S468N	S124N	3*	Since 2009^**+**^
A1429G	I477V	I133V	1*	2009–2016
A1523G	E508G	E164G	1	2015–2016
A1537G	N513D	N169D	1	2013
G1546A	E516K	E172K	3*	Since 2009^**+**^
C1656A	F552L	F208L	1	2009

The numbering of the aa exchanges is presented based on the position on the HA ORF as well as in the HA1 and HA2 domains. SP = signal peptide.

* = Substitution occurs in Palestinian A(H1N1)pdm09 isolates from vaccinated participants.

^**+**^ = Substitution occurs also in A/Michigan/45/2015(H1N1), H1N1 vaccine component for the years 2017/2018 and 2018/2019.

**Table 2:** Non-synonymous substitutions in the NA gene of Palestinian A(H1N1)pdm09 isolates (n = 10).

nt NA	aa NA	Occurrence in Palestinian Sequences	Circulation of Substitution
A11C	N4T	1*	2009–2016
A83G	N28S	1	2009–2016
A100G	I34V	4*	Since 2009^**+**^
C118A	L40I	4*	Since 2010^**+**^
A131G	N44S	4*	Since 2009^**+**^
G199A	V67I	3	Since 2009^**+**^
C236T	S79L	3	Since 2009
T244C	S82P	1	Since 2009
A599G	N200S	11*	Since 2009^**+**^
A631T	I211L	1	2010, 2015
G721A	V241I	11*	Since 2009^**+**^
G790A	V264I	8*	Since 2009^**+**^
T810A	N270K	8*	Since 2010^**+**^
A961G	I321V	10*	Since 2009^**+**^
G993T	K331N	1*	2009
A1052T	Y351F	11*	Since 2011^**+**^
T1094C	I365T	1	2009–2016
C1107A	N369K	11*	Since 2009^**+**^
C1158A	N386K	10*	Since 2009^**+**^
A1294G	K432E	9*	Since 2009^**+**^

* = Substitution occurs in Palestinian A(H1N1)pdm09 isolates from vaccinated participants.

^**+**^ = Substitution occurs also in A/Michigan/45/2015(H1N1), H1N1 vaccine component for the years 2017/2018 and 2018/2019.

### Substitutions in the HA and NA of Palestinian A(H3N2) isolates

23 non-synonymous substitutions were identified in the HA genes of the Palestinian influenza A virus subtype A(H3N2) (Table 3). 18 of the 23 substitutions occurred in the HA1 domain and five in the HA2 domain, respectively. HA1 substitutions D53N, E62K, N121K, N121A, N122D, N128A, N128T, R142G, N144S, N144R, N145S, L157S, F159Y, K160T, P198S, N225D, and Q311H were located within antigenic epitopes of the HA gene. 26 synonymous substitutions were also detected in the Palestinian HA genes of the A(H3N2) isolates (S4 Table). Eight non-synonymous (Table 4) and 21 synonymous substitutions (S5 Table) occurred in the NA genes of Palestinian A(H3N2) isolates.

**Table 3:** Non-synonymous substitutions in the HA gene of A(H3N2) Palestinian isolates (n = 12).

nt HA	aa HA	aa HA1 HA2	Occurrence in Palestinian Sequences	Circulation of Substitution
C55A	L19I	L3I	11*	Since 2012+^
G205A	D69N	D53N	1	Since 2012
G232A	E78K	E62K	1	Since 2012
C411A	N137K	N121K	1	Since 2012^
A412G	N138D	N122D	1	Since 2013
A430G	N144A	N128A	1	Since 2012
A431C	N144T	N128T	11*	Since 2012+^
A472G	R158G	R142G	1	Since 2012
A479G	N160S	N144S	10*	Since 2012+^
T480G	N160R	N144R	1	Since 2015
A482G	N161S	N145S	11*	Since 2012+^
T518C	L173S	L157S	1	Since 2012
T524A	F175Y	F159Y	11*	Since 2012+^
A527C	K176T	K160T	11*	Since 2013+
C640T	P214S	P198S	12*	Since 2012+^
G682A	A228T	A212T	1	Since 2012
A721G	N241D	N225D	11*	Since 2012+^
A981T	Q327H	Q311H	11*	Since 2013+
G1087A	V363K	V18K	1	2013–2015
T1088A				
A1462G	K488E	K143E	2*	Since 2012
G1513A	D505N	D160N	11*	Since 2012+^
G1633A	V545I	V200I	4*	Since 2013
A1651G	I551V	I206V	2*	Since 2012

The numbering of aa substitutions is presented by the position on the HA ORF as well as in the HA1 and HA2 domains.

* = Substitution occurs in Palestinian A(H3N2) isolates from vaccinated participants.

+ **=** Substitution occurs also in A/SWITZERLAND/9715293/2013(H3N2), vaccine strain 2015/2016

^ = Substitution occurs also in A/HONG KONG/4801/2014(H3N2), vaccine strain 2016/2017 and 2017/2018.

**Table 4:** Non-synonymous substitutions in the NA gene of the Palestinian A(H3N2) isolates (n = 7).

nt NA	aa NA	Occurrence in Palestinian Sequences	Circulation of Substitution
A374G	D125G	1	Since 2012
A449G	H150R	7*	Since 2012+^
G661A	E221N	1	2014–2016
A663T A663C	E221D	5*	Since 2012+^
C800A	T267K	6*	Since 2012+^
C987A	N329K	1	Since 2012
A1138G	I380V	5*	Since 2013+^
T1175C	I392T	1	Since 2012

* = Substitution occurs in Palestinian A(H3N2) isolates from vaccinated participants.

+ **=** Substitution occurs also in A/SWITZERLAND/9715293/2013(H3N2), vaccine strain 2015/2016

^ = Substitution occurs also in A/HONG KONG/4801/2014(H3N2), vaccine strain 2016/2017 and 2017/2018.

### Phylogenetic analysis of Palestinian A(H1N1)pdm09 isolates

**Fig 3:**
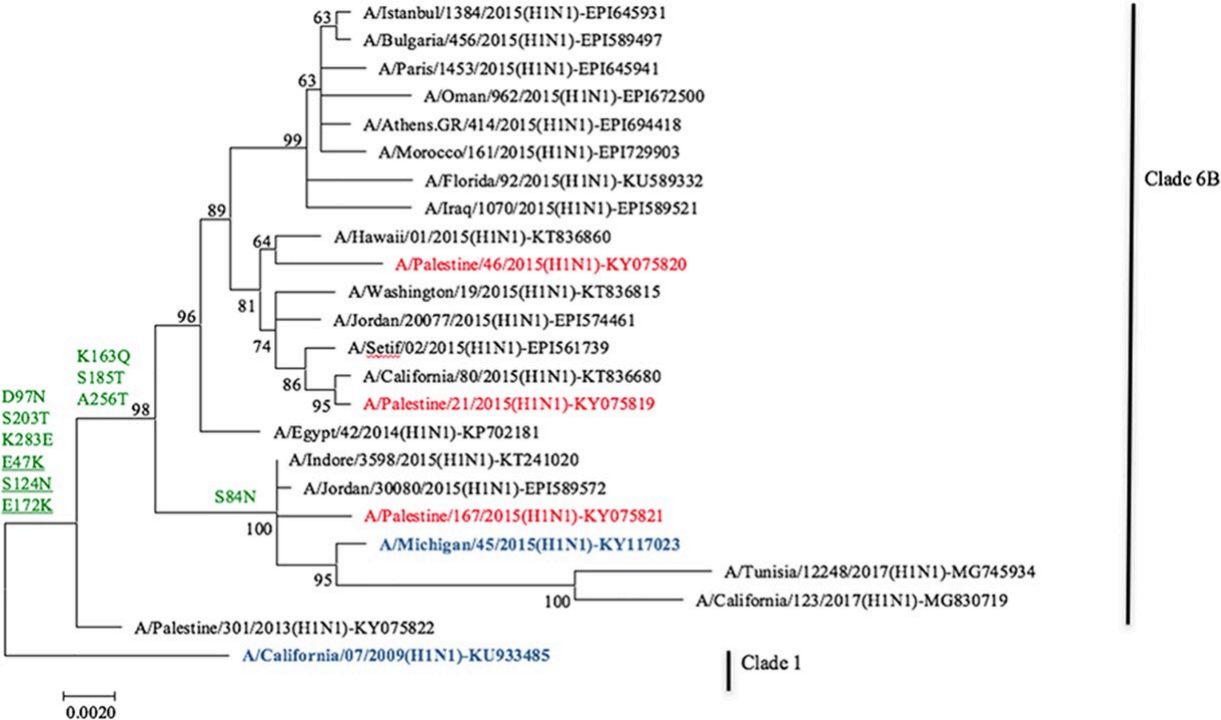
Phylogenetic analysis of the HA gene segment of Palestinian influenza A(H1N1)pdm09 isolates. Palestinian HA sequences (in red) were compared with those from regional and international isolates, as well as vaccine strain of the season 2014/2015 and the current vaccine strain (in dark blue). NCBI and GISAID accession numbers are indicated for all archived sequences. HA1 aa substitutions are in green and HA2 aa substitutions are in green and underlined.

Both HA and NA sequences of Palestinian A(H1N1)pdm09 isolates showed percent identity around 97–98% with A/California/7/2009, the H1N1 vaccine component between 2011 and 2016, which includes our sampling season. Percent identity was however ~99% for HA and >99% for NA with A/Michigan/45/2015(H1N1)pdm09, the H1N1 vaccine component since season 2016/2017. All three Palestinian isolates with full length HA gene carried substitutions D97N, K163Q, S185T, S203T, A256T, K283E in the HA1 domain and E47K, S124K, E172K in the HA2 domain and consequently fell into clade 6B (Table 1 and Fig 3), respectively. Phylogenetic analysis showed that Palestinian A(H1N1)pdm09 isolates did not necessarily cluster with regional isolates (Fig 3). Palestinian KY075819 was almost identical with an isolate from California of the same year (KT836680), Palestinian KY075820 clustered with an isolate from Hawaii (KT836860), and Palestinian KY075821 clustered with an isolate from India (KT241020) and Jordan (EPI589572) on the same branch with A/Michigan/45/2015(H1N1).

### Phylogenetic analysis of Palestinian A(H3N2) isolates

**Fig 4:**
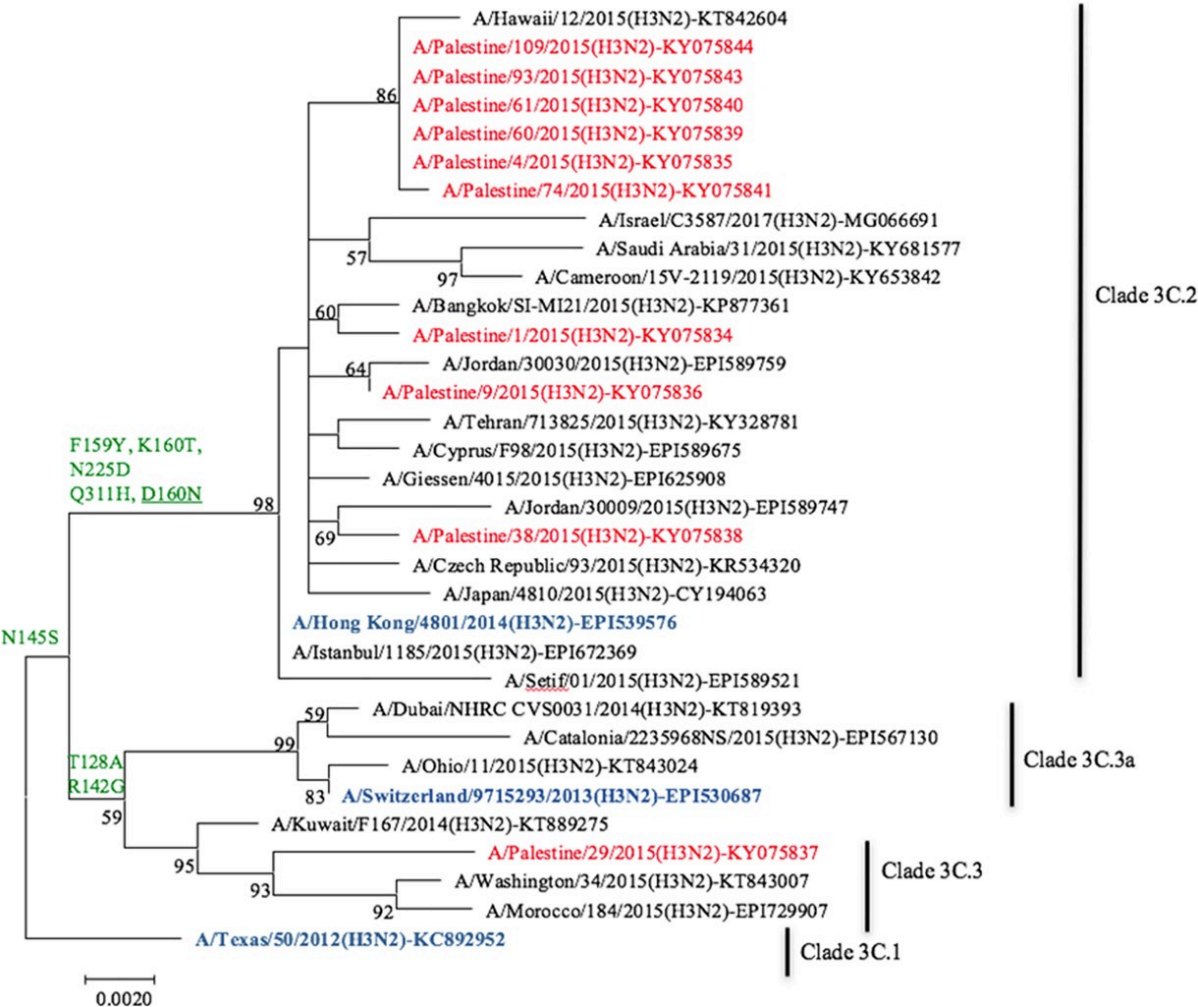
Phylogenetic analysis of the HA gene segment of Palestinian influenza A(H3N2) isolates. Palestinian HA sequences (in red) were compared with those from regional and international isolates, as well as vaccine strain of seasons 2014/2015-2017/2018 (in dark blue). NCBI and GISAID accession numbers are indicated for all archived sequences. HA1 aa substitutions are in green and HA2 are in green and underlined.

Percent identities between 11 of the 12 HA sequences of Palestinian A(H3N2) and A/Texas/50/2012(H3N2), vaccine component of our isolation season 2014/2015, ranged from 97.4–98.4%. However, it was >99% with A/Hong Kong/4801/2014(H3N2), the vaccine component of seasons 2016/2017 and 2017/2018. Ten of these 11 Palestinian sequences exhibited substitutions L3I, N144S, N145S, F159Y, K160T, N225D, Q311H in the HA1 domain, and D160N in the HA2 (Table 3) designating them to subclade 3C.2a. One (KY075834) of these 11 Palestinian sequences possessed all above substitutions but N144S and therefore fell into clade 3C.2. The twelfth Palestinian HA sequence (KY075845) contained N128A, R142G, N145S substitutions, and therefore fell into clade 3C.3. Ten (with full length HA gene) of the 12 HA sequences of Palestinian H3N2 isolates subjected to phylogenetic analysis (Fig 4) clustered together or/and with isolates from Bangkok (KP877361), Hawaii (KT842604), and Jordan (EPI589747 and EPI589759). Palestinian KY075845 clustered with an isolate from Washington (KT843007).

In regards to NA sequences of Palestinian H3N2 isolates, all, including the NA of the isolate to which KY075845 belongs, showed percent identity >99% with A/Switzerland/9715293/2013(H3N2), vaccine strain of 2015/2016.

## Discussion

Human influenza A viruses continue to show significant impacts on public health and global economy by causing annual outbreaks and occasional pandemics. Though from 2015, hitherto, this is the first molecular epidemiology study on influenza A circulating in Palestine and is actually one of the few reports in that regard from and on the Middle East [19, 20].

Our study demonstrated that the rate of infection with IAV among the different Palestinian age groups did not differ significantly and ranged from 20%-30%. The highest infection rate was detected in the ≤1-year-olds group, which in this study, were subjects that were either hospitalized, or visited a private clinic. Indeed, infants are considered to be at a high risk for influenza infection and associated with hospitalization (reviewed in [21]). Nevertheless, Fell et al. [21] argue, that there is a lack of studies in this regard and the majority of the data was from the USA. On the other hand, a comprehensive Flu Watch Group cohort study [22], concluded that influenza A infected 24% or 22% of unvaccinated people in the season preceding pandemic or during pandemic season, and that infection rates were highest in children, which generally agrees with our findings.

Our study elucidated that both A(H1N1)pdm09 and A(H3N2) were co-circulating in Palestine between February and May 2015 without an overall predominance of one subtype or the other. This finding was in accordance or was partially in accordance with regional studies from Lebanon, Iran, and Tunisia [19, 23, 24], which were performed during the same influenza season of our sampling. However, our findings did not agree with WHO weekly influenza surveillance reports of 2015 for season 2014/2014, which revealed a predominance of A(H1N1)pdm09 subtype in the Middle East [25]. Hence, we reviewed the Flumart Outputs of the WHO FluNet database [26] for the Middle Eastern and North African countries for the exact period of our sampling. Reasonable data (IAV subtyped cases ≥ 50 over 8 weeks of our sampling time) were available from Algeria, Egypt, Iran, Israel, Jordan, Oman, Qatar, Tunisia and Turkey. While no one single A(H3N2) case was indicated from Qatar, the A(H1N1)pdm09 reported cases by far outnumbered A(H3N2) cases from Algeria, Egypt, Jordan, Oman, Tunisia and Turkey. The percentages of subtyped A(H3N2) and A(H1N1)pdm09 cases from Iran were similar to our results and to those of Moasser et al. [24] showing no predominance of one subtype. Interestingly, A(H3N2) was predominant in Israel according to the WHO Flumart Outputs [26] and a recent Israeli study [27], which was in line with surveillance reports from the northern hemisphere for season 2014/2015, including the USA, Canada and Europe, [8, 25, 28–30]. The discrepancies between our findings and regional reports, including the WHO influenza surveillance report in regard to subtype dominance cannot be attributed to our study setting in regard to targeted study sample, as both subtypes were present in all groups of our study sample; hospitalized patients, outpatients, kindergarten children, private clinic patients, and private individuals. From another aspect, our study revealed that the odds of being infected with A(H1N1)pdm09 among adults was higher than in infants and children ≤ 6-year-old, while the odds of being infected with A(H3N2) was higher in infants and children ≤ 6-year-old. Nevertheless, The relationship between age and infection with a specific influenza A subtype is a matter of inconsistencies between different reports, which may vary with season, epidemic timing, lack of pre-existing immunity against novel A(H3N2), and study settings [31, 32]. In our case, this result could be attributed to the limitation of our study in regard to the number of samples collected in these different groups and the months of collection.

Seventeen non-synonymous substitution events were recorded in the HA1 and HA2 aa sequences of the Palestinian A(H1N1)pdm09. The Palestinian A(H1N1)pdm09 isolates fell into clade 6B. A(H1N1)pdm09 with clade 6B were predominant for season 2014/2015 as indicated by the WHO [9], regional [24], and International reports [33–35]. Most of the 6B clade-defining substitutions evolved during the last years and were reported earlier in some studies before being attributed to the new clades. S185T was previously reported in isolates from Tunisians with severe influenza A infection [36]. S203T contributes to antigenic drift [37] and is a common variant marker within an outbreak [12]. Substitution S84N, found in HA gene of one Palestinian isolate is one of the substitutions defining the novel genetic subclade 6B.1 [33, 34, 38]. Substitution I321V is also a common variant marker within outbreaks [12] and was found in all Palestinian isolates. E47K, S124K, and E172K substitutions located in the HA2 do not only define clade 6B; they were reported, along with other substitutions, to increase HA stability through generation of favorable inter- and intramonomer interactions [39]. A variant with E47K was also shown to induce infection in ferrets [40].

We detected 20 non-synonymous substitutions in the NA sequences from 10 Palestinian A(H1N1)pdm09 isolates. All of these substitutions were circulating in NCBI- and GISAID-archived sequences. Some of these substitutions were detected in oseltamivir-resistant strains, but do not confer resistance per se, such as N44S, N200S, V241I, and N369K [41]. Substitutions S79L and K331N, occurred independently in three and in one Palestinian NA sequences, were studied thoroughly by Ilyushina et al. [42], who demonstrated that S79L reduced the activity of the NA enzyme, while the K331N was associated with evidently increased resistance to IFN-λ1. Substitutions V241I, N369K, N386K and K432E, detected in all Palestinian A(H1N1)pdm09 NA sequences, may alter the binding affinity between oseltamivir and NA, and consequently affect susceptibility of A(H1N1)pdm09 strains to oseltamivir [41]. Substitution I365T, which occurred in one Palestinian sequence, was related to virulence and antigenic drift/escape mutant [12]. Substitution N44S detected in four Palestinian samples created a new potential N-glycosylation site at position 42, while N386K, detected in all Palestinian isolates, removes a potential N-glycosylation site at position 386, both cases may affect antigenic properties of the strain [12].

Ten Palestinian 2015 A(H3N2) isolates fell into subclade 3C.2a, one into clade 3C.2, and one into clade 3C.3, respectively. This result was in accordance with the WHO, regional and international reports of the same season [9, 24, 28, 35, 43–45]. Interestingly, the neuraminidase gene sequences of all Palestinian A(H3N2) were closely related to A/Switzerland/9715293/2013(H3N2), subclade 3C.3a, which was a distinctive finding observed for the A(H3N2) isolates from 2015 [35, 45]. Each of non-synonymous substitutions N144S, N144R and N122D remove a potential N-glycosylation site, while K160T and N128T create a new potential N-glycosylation, with the consequences of affecting antigenic properties [12]. Some of the substitutions (N121K, P198S, L157S), which were detected only in the single Palestinian 3C.3 HA sequence (KY075845) are of significant interest. N121K became dominant in 2017 and was detectd in isolates from severe influenza cases in Hong Kong in 2017 [46] and was attributed to lower vaccine effectiveness in clades 3C.2a and 3C.2a1 [47]. Substitutions P198S and L157S had been related to virulence and antigenic drift/escape mutant [12].

Of the eight non-synonymous substitutions recorded in the NA sequences of the Palestinian A(H3N2), H150R, E221N and E221D, and N329 are positions where substitutions had been related to antigenic drift/escape mutants [12]. N329K removes a potential N-glycosylation site at position 329, which may affect antigenic properties of this strain [12].

Finally, we detected one single synonymous substitution in the HA signal peptide region of each subtype; T30C: Y10Y in one A(H1N1)pdm09 isolate and T18C: A6A in one A(H3N2) isolate (S2 and S4 Tables). Although none of these synonymous substitutions were addressed in the literature, *in vitro* experiments in a recent study revealed that synonymous mutations at the beginning of the HA gene may affect viral fitness [48].

The emergence of IAV substitutions/mutations should be carefully monitored for public health concerns, as detailed above, many amino acid substitutions evolved during outbreaks and were only detected in few cases before becoming dominant few years later. Therefore, we believe that our study contributes to the international efforts by providing epidemiological and molecular biological insights into influenza A viruses circulating in Palestine and advocate for comprehensive annual typing and mutation analyses of Influenza A in Palestine and the region.

## Supporting information

S1 Table Accession numbers of archived sequences used for substitution analysis. (DOCX)

S2 Table Synonymous substitutions in the H1 gene of Palestinian H1N1 isolates (N = 3). The aa substitutions are presented by the position on HA gene as well as HA1 and HA2 subunits. 2015^**+**^ = 2015 isolates including A/Michigan/45/2015(H1N1), the H1N1 vaccine component for the years 2017/2018 and 2018/2019.
(DOCX)

S3 Table Synonymous substitutions in the NA gene of the Palestinian H1N1 isolates (n = 10). 2015^**+**^ = 2015 isolates including A/Michigan/45/2015(H1N1), the H1N1 vaccine component for the years 2017/2018 and 2018/2019.
(DOCX)

S4 Table Synonymous substitutions in the HA gene of the Palestinian H3N2 sequences (n = 12). The aa substitutions are presented by their position on the HA gene and by HA1 and HA2 subunits. + **=** Substitution occurs also in A/Switzerland/9715293/2013(H3N2), vaccine strain 2015/2016, ^ = Substitution occurs also in A/Hong Kong/4801/2014(H3N2), vaccine strain 2016/2017 and 2017/2018.
(DOCX)

S5 Table Synonymous substitutions in the NA gene of the Palestinian H3N2 sequences (n = 7). + **=** Substitution occurs also in A/Switzerland/9715293/2013(H3N2), vaccine strain 2015/2016, ^ = Substitution occurs also in A/Hong Kong/4801/2014(H3N2), vaccine strain 2016/2017 and 2017/2018.
(DOCX)
