# Supplementary material for: Molecular epidemiology survey and characterization of human influenza A viruses circulating among Palestinians in East Jerusalem and the West Bank in 2015
Source: PLoS One. 2019 Mar 8;14(3):e0213290. doi: 10.1371/journal.pone.0213290 (PMC6407757; doi:10.1371/journal.pone.0213290)
Supplement: S1 Table — (DOCX) [file pone.0213290.s001.docx]

**S1 Table. Accession numbers of archived sequences used for substitution analysis.**

**A. Archived sequences used for H1 substitution analysis.**

**NCBI:** KP702181, KU589332, KT241020, KT836680, KT836860, KT836815, MG830719**,** MG745934**,** KU933485, KY117023,

**GISAID:** EPI645931, EPI589497, EPI645941, EPI672500, EPI694418, EPI729903, EPI589521, EPI574461, EPI561739, EPI589572

**B. Archived sequences used for N1 substitution analysis**.

**NCBI:** KT836766, KT836484, KT836688, KU310642, KU509745, KU695616, MG830725, MG830744, KU589334, GQ377078, KY117025, KY075826

**GISAD:** EPI561740, EPI574462, EPI589573, EPI589522, EPI589498, EPI645932, EPI672503, EPI645942, EPI694419, EPI729111

**C. Archived sequences used for H3 substitution analysis:**

**NCBI:** KT819393**,** KT889275, KT842604, KU591614, KY681577, KT843007, CY194063, KR534320, KP877361, KT843024, MG066691, KC892952, KY653842, KY328781

**GISAID:** EPI589759, EPI589675, EPI625908, EPI589747, EPI672369, EPI589521, EPI567130, EPI729907, EPI539576, EPI530687

**D. Archived sequences used for N2 substitution analysis**

**NCBI:** KT819397, KT888874, KT842606, KU591932, KT842972, KP877393, KT843333, KY681627, KC892237, KY653843, KY328818

**GISAID:** EPI589748, EPI625907, EPI589676, EPI589760, EPI672370, EPI563359, EPI567131, EPI729908, EPI530688, EPI539577
