# Supplementary material for: Molecular epidemiology survey and characterization of human influenza A viruses circulating among Palestinians in East Jerusalem and the West Bank in 2015
Source: PLoS One. 2019 Mar 8;14(3):e0213290. doi: 10.1371/journal.pone.0213290 (PMC6407757; doi:10.1371/journal.pone.0213290)
Supplement: S2 Table — The aa substitutions are presented by the position on HA gene as well as HA1 and HA2 subunits. 2015+ = 2015 isolates including A/Michigan/45/2015(H1N1), the H1N1 vaccine component for the years 2017/2018 and 2018/2019. (DOCX) [file pone.0213290.s002.docx]

**S2 Table. Synonymous substitutions in the H1 gene of Palestinian H1N1 isolates (N=3).** The aa substitutions are presented by the position on HA gene as well as HA1 and HA2 subunits. 2015**^+^**= 2015 isolates including A/Michigan/45/2015(H1N1), the H1N1 vaccine component for the years 2017/2018 and 2018/2019.

| **nt**  **HA** | **aa**  **HA** | **aa**  **HA1/**  **HA2** | **Occurrence in**  **Palestinian Sequences** | **Circulation of Substitution** |
| --- | --- | --- | --- | --- |
| T30C | Y10Y | SP | 1 | After 2013 |
| A150G | L50L | L33L | 1 | 2015, 2017 |
| G168A | G56G | G39G | 1 | 2014, 2015, 2017 |
| A315G | G105G | G88G | 1 | No |
| G390A | R130R | R113R | 2 | 2014, 2015 |
| T465C | H155H | H138H | 3 | After 2013 |
| T492C | N164N | N147N | 1 | 2015, 2017 |
| C591T | H197H | H180H | 1 | 2017 |
| T648C | V215V | V198V | 2 | 2015 |
| A966G | K322K | K305K | 3 | After 2011 |
| A972G | V324V | V307V | 2 | 2014, 2015 |
| G1017A | P339P | P322P | 2 | 2014, 2015 |
| T1041C | F347F | F3F | 3 | After 2011 |
| C1047A | A349A | A5A | 1 | No |
| G1173A | K391K | K47K | 2 | 2014, 2015 |
| A1230G | V410V | V66V | 3 | After 2011 |
| C1249T | L417L | L73L | 2 | 2013, 2014, 2015 |
| C1320T | A440A | A96A | 1 | No |
| T1327C | L443L | L99L | 2 | 2014, 2015 |
| C1362T | Y454Y | Y110Y | 1 | 2015**^+^**, 2017 |
| G1395A | K465K | K121K | 1 | 2015**^+^**, 2017 |
| T1467C | D489D | D145D | 1 | 2015, 2017 |
| G1473A | T491T | T147T | 1 | 2017 |
| G1578A | R526R | R182R | 2 | 2014, 2015 |
| T1581C | I527I | I183I | 1 | 2015, 2017 |
| T1608C | T536T | T192T | 1 | No |
| T1653C | S551S | S207S | 3 | After 2011 |
