# Supplementary material for: Molecular epidemiology survey and characterization of human influenza A viruses circulating among Palestinians in East Jerusalem and the West Bank in 2015
Source: PLoS One. 2019 Mar 8;14(3):e0213290. doi: 10.1371/journal.pone.0213290 (PMC6407757; doi:10.1371/journal.pone.0213290)
Supplement: S3 Table — 2015+ = 2015 isolates including A/Michigan/45/2015(H1N1), the H1N1 vaccine component for the years 2017/2018 and 2018/2019. (DOCX) [file pone.0213290.s003.docx]

**S3 Table. Synonymous substitutions in the NA gene of the Palestinian H1N1 isolates (n=10).** 2015**^+^**= 2015 isolates including A/Michigan/45/2015(H1N1), the H1N1 vaccine component for the years 2017/2018 and 2018/2019.

| **nt**  **NA** | **aa**  **NA** | **Occurrence in Palestinian Sequences** | **Circulation of Substitution** |
| --- | --- | --- | --- |
| C27A | T9T | 2 | 2015 |
| A81G | G27G | 3 | 2014, 2015 |
| G123A | G41G | 4 | 2014, 2015**^+^**, 2017 |
| T162C | I54I | 1 | No |
| T225C | A75A | 1 | No |
| C375T | S125S | 4 | 2014, 2015^+^, 2017 |
| C627T | N209N | 10 | 2013-2015^+^ |
| A660G | R220R | 10 | 2014, 2015+, 2017 |
| T666C | N222N | 1 | No |
| G684A | E228E | 1 | 2015 |
| T708C | G236G | 1 | No |
| T720C | T240T | 9 | 2011-2015+, 2017 |
| T720A | T240T | 1 | No |
| C729T | T243T | 1 | No |
| C753T | A251A | 1 | No |
| A783G | G261G | 1 | No |
| T846C | Y282Y | 8 | 2015^+^, 2017 |
| G1044A | G348G | 10 | 2010-2017+ |
| T1059C | Y353Y | 10 | 2010-2017+ |
| C1134T | N378N | 10 | 2011-2015+, 2017 |
| A1149T | T383T | 9 | 2014, 2015+-2017 |
