# Supplementary material for: Molecular epidemiology survey and characterization of human influenza A viruses circulating among Palestinians in East Jerusalem and the West Bank in 2015
Source: PLoS One. 2019 Mar 8;14(3):e0213290. doi: 10.1371/journal.pone.0213290 (PMC6407757; doi:10.1371/journal.pone.0213290)
Supplement: S4 Table — The aa substitutions are presented by their position on the HA gene and by HA1 and HA2 subunits. + = Substitution occurs also in A/Switzerland/9715293/2013(H3N2), vaccine strain 2015/2016, ^ = Substitution occurs also in A/Hong Kong/4801/2014(H3N2), vaccine strain 2016/2017 and 2017/2018. (DOCX) [file pone.0213290.s004.docx]

**S4 Table. Synonymous substitutions in the HA gene of the Palestinian H3N2 sequences (n=12).** The aa substitutions are presented by their position on the HA gene and by HA1 and HA2 subunits. +**=** Substitution occurs also in A/Switzerland/9715293/2013(H3N2), vaccine strain 2015/2016, ^= Substitution occurs also in A/Hong Kong/4801/2014(H3N2), vaccine strain 2016/2017 and 2017/2018.

| **nt** | **aa**  **HA** | **aa**  **HA1/**  **HA2** | **Occurrence in**  **Palestinian Sequences** | **Circulation of Substitution** |
| --- | --- | --- | --- | --- |
| T18C | A6A | SP | 1 | 2015, 2016**^+^** |
| C90T | C30C | C14C | 1 | No |
| G138A | T46T | T30T | 1 | 2015 and after |
| C144T | D48D | D32D | 1 | No |
| A171G | E57E | E41E | 11 | 2012-2016**^+^**, 2017**^+^** |
| T213C | P71P | P55P | 1 | 2015 |
| A231G | G77G | G61G | 1 | No |
| C244T | L82L | L66L | 1 | No |
| G273A | Q91Q | Q75Q | 1 | No |
| C285T | F95F | F79F | 12 | 2012-2016**^+^**, 2017**^+^** |
| C396T | G132G | G116G | 1 | No |
| T480C | N160N | N144N | 1 | No |
| C600T | H200H | H184H | 7 | 2015 |
| T663C | S221S | S205S | 1 | 2015 |
| G693A | P231P | P215P | 11 | 2015, 2016**^+^**, 2017**^+^** |
| T870A | I290I | I274I | 7 | 2015 |
| G978A | K326K | K310K | 1 | No |
| G1011A | R377R | R32R | 12 | 2012-2016**^+^**, 2017**^+^** |
| G1260A | G420G | G75G | 11 | 2015 and after |
| T1296A | T432T | T87T | 1 | 2014, 2015 |
| T1368A | T456T | T111T | 4 | No |
| C1437A | G479G | G134G | 11 | Most 2015 and after |
| C1491A | I497I | I152I | 11 | 2015 and after |
| A1518G | V506V | V161V | 11 | 2015 and after |
| A1533T | A511A | A166A | 1 | 2015 |
| G1683A | R561R | R216R | 1 | 2016**^+^**, 2017**^+^** |
