# Supplementary material for: Molecular epidemiology survey and characterization of human influenza A viruses circulating among Palestinians in East Jerusalem and the West Bank in 2015
Source: PLoS One. 2019 Mar 8;14(3):e0213290. doi: 10.1371/journal.pone.0213290 (PMC6407757; doi:10.1371/journal.pone.0213290)
Supplement: S5 Table — + = Substitution occurs also in A/Switzerland/9715293/2013(H3N2), vaccine strain 2015/2016, ^ = Substitution occurs also in A/Hong Kong/4801/2014(H3N2), vaccine strain 2016/2017 and 2017/2018. (DOCX) [file pone.0213290.s005.docx]

**S5 Table. Synonymous substitutions in the NA gene of the Palestinian H3N2 sequences (n=7).** +**=** Substitution occurs also in A/Switzerland/9715293/2013(H3N2), vaccine strain 2015/2016, ^= Substitution occurs also in A/Hong Kong/4801/2014(H3N2), vaccine strain 2016/2017 and 2017/2018.

| **nt**  **NA** | **aa**  **NA** | **Occurrence in Palestinian Sequences** | **Circulation of Substitution** |
| --- | --- | --- | --- |
| C84T | I28I | 1 | 2014, 2015 |
| C129T | N43N | 1 | No |
| C202T | L68L |  | 2013-2016+, 2017+ |
| G204A | L68L | 2 | 2014, 2015 |
| C339T | D113D | 1 | No |
| G408A | Q136Q | 1 | 2014, 2015 |
| G429A | V143V | 1 | No |
| A447G | V149V | 3 | 2015 |
| T501C | F167F | 7 | 2014-2016+, 2017+ |
| G585A | T195T | 1 | No |
| C675T | T225T | 1 | No |
| G678A | Q226Q | 1 | No |
| C825T | V275V | 1 | 2014, 2015 |
| A888G | K296K | 1 | 2014, 2015 |
| A981G | R327R | 1 | No |
| T1065C | D355D | 1 | 2014 |
| A1089G | G363G | 7 | 2015, 2016+, 2017+ |
| C1155T | N385N | 1 | 2011, 2014, 2015 |
| G1170A | L390L | 1 | Novel |
| A1239G | E413E | 1 | Novel |
| C1347A | T449T | 1 | No |
